# Skin Gambling Contributes to Gambling Problems and Harm After Controlling for Other Forms of Traditional Gambling

**DOI:** 10.1007/s10899-022-10111-z

**Published:** 2022-02-25

**Authors:** Nancy Greer, Matthew Rockloff, Nerilee Hing, Matthew Browne, Daniel L. King

**Affiliations:** 1 Experimental Gambling Research Laboratory, School of Health, Medical and Applied Sciences, CQUniversity Australia, Melbourne, VIC Australia; 2 Experimental Gambling Research Laboratory, School of Health, Medical and Applied Sciences, CQUniversity Australia, Bundaberg, QLD Australia; 3 College of Education, Psychology, and Social Work, Flinders University, Adelaide, Australia

**Keywords:** Esports betting, Gambling, Skin gambling, Loot box, Gambling problems, Gambling harm

## Abstract

Esports betting and skin gambling involve betting on the outcomes of video game competitions and/or using virtual currencies for betting. The present study evaluated a conceptual model linking video game involvement, video-game related gambling, traditional gambling, and gambling problems and harm. Data were collected via a cross-sectional online survey with 737 participants aged 18 + who engaged in esports cash betting (n = 576), esports skin betting (n = 184), or skin gambling on games of chance (n = 330). The findings highlighted the distinctly different relationships esports cash betting versus skin gambling had with traditional gambling involvement and harmful gambling. Gambling with skins on games of chance was predictive of gambling problems and gambling harm after controlling for participation in traditional gambling (OR = 1.32 and 1.17 respectively). Whereas betting on esports with cash was associated with betting on a variety of other forms of gambling, and there was no unique contribution to problems and harm over and above participation on these other forms (e.g., EGMs, sports betting). Skin gambling is directly implicated in gambling problems and harm, whereas cash betting on esports is only indicative of interest in many forms of potentially harmful gambling. Greater research attention to skin gambling is warranted, and particularly with respect to its role as a virtual currency more easily accessible for gambling.

**Supplementary Information:**

The online version contains supplementary material available at 10.1007/s10899-022-10111-z.

## Introduction

Video games have created new opportunities to spend money and gamble because of two recent innovations: (1) esports (professional video gaming competitions), and (2) skins (in-game digital items, such as visual enhancements to characters, that have a marketplace monetary value). These two innovations, which by themselves are not necessarily intended to enable gambling, nevertheless have enabled new gambling products. Wagering operators accept cash bets on esports, just like on other sports. Additionally, skin gambling operators accept skins as a digital currency for betting on esports and simple games of chance such as coin-flips or roulette (Greer et al., 2019; Grove, 2016). That is, gambling providers and unregulated third-party websites have exploited these opportunities to provide new gambling products and a new currency, skins, with which to gamble on traditionally conceived gambling activities.

Esports spectators, esports players, and video gamers are the people most often exposed to opportunities to bet on esports, as well as with skins, through their video game and esports involvement. Engagement with these new products may prompt gamers to try traditional forms of gambling, such as sports betting or electronic gaming machines (EGMs). These “video game-related gamblers” (i.e., esports bettors and skin gamblers) may then experience gambling problems or harm directly from these new video-game-related gambling products, from traditional gambling activities, or from a combination of both; such as using skins as a digital currency for gambling.

**Fig. 1:**
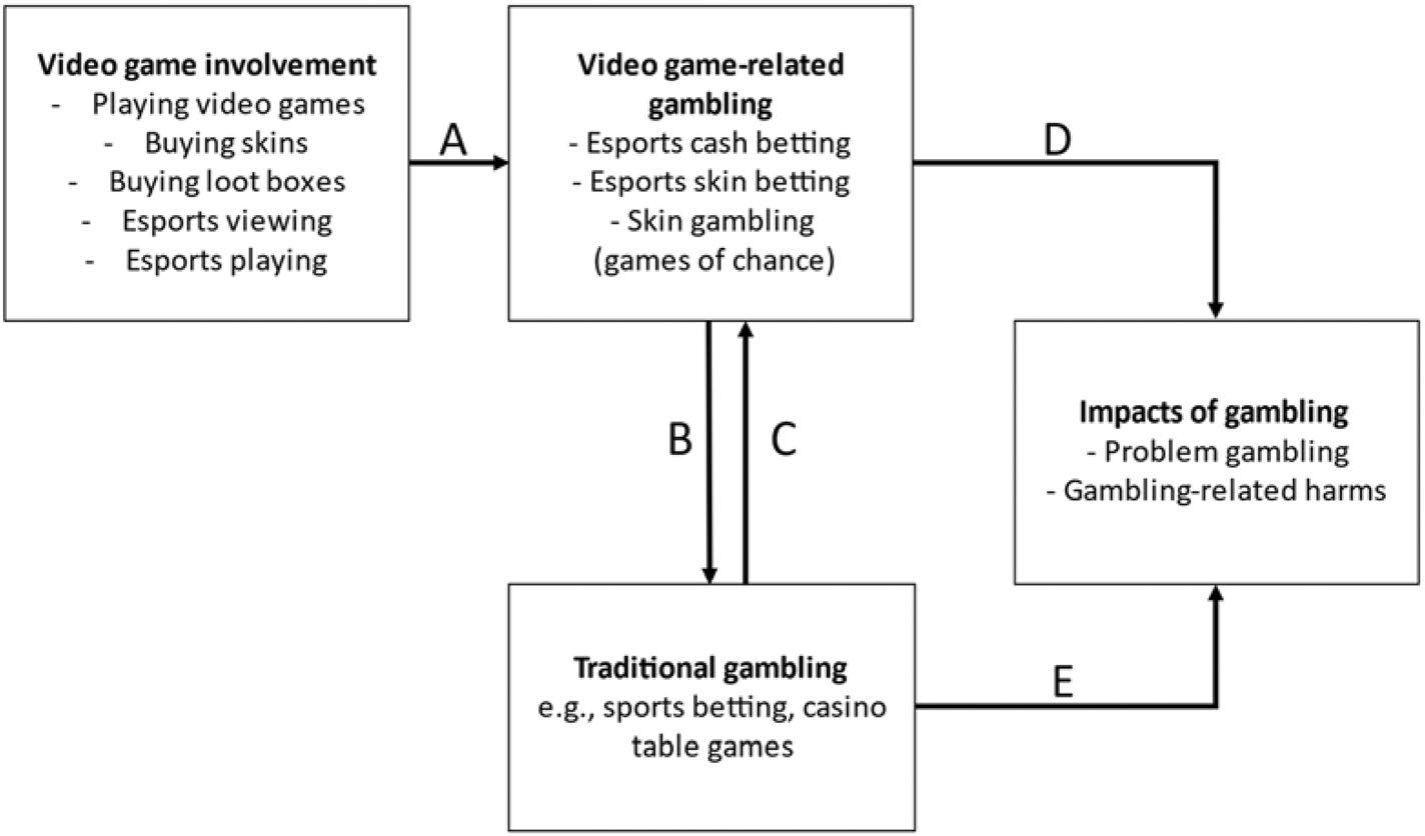
A conceptual path model of the relationships between video game-related gambling and video game involvement (A), traditional gambling (B, C), and the impacts of gambling (D, E)

It may be premature to state that a “gamblification” of video games and esports is creating a new pathway for youth to experience gambling problems and harm (Brock & Johnson, 2021; Delfabbro & King, 2020; King et al., 2019). However, the potential for some video gaming activities and video game-related gambling to increase the likelihood of transitioning to gambling and attendant gambling problems needs to be explored (Kim & King, 2020). Specifically, it is important to understand where the greatest risks for involvement in video game-related gambling are, and how much (if any) later experiences of gambling-related problems and harm can be attributed to esports betting or skin gambling, as opposed to traditional gambling activities. The current research aims to test the conceptual model outlined in Fig. 1. The conceptual model is based on hypothesised logical connections, explained in more detail below, between behaviors and outcomes to simplify relationships for testing.

### Video Gaming Involvement: Primers for Video Game-Related Gambling (A)

Path A in Fig. 1 proposes that involvement in specific video game activities precedes involvement in video game-related gambling. *Video game involvement* can include: (1) playing video games, (2) buying skins or loot boxes, (3) watching esports, and (4) playing esports competitively. Increased involvement in these activities may increase the likelihood of engagement in video game-related gambling: In this model *video game-related gambling* encompasses: (1) esports cash betting with money or cryptocurrency, (2) esports skin betting, and (3) skin gambling on games of chance.

*Playing video games.* Firstly, in path explores the relationship between *playing video games* and video-game related gambling activities *esports cash betting*, *esports skin betting*, and *skin gambling on games of chance*. Conceptually greater involvement in video gaming may increase the likelihood of being exposed to esports, skins, and the associated gambling opportunities; albeit most likely only for video games which have links to esports or which feature skins. Furthermore, some video game may have an online ecosystem (e.g., online communities, online influencers, sponsors, and marketing) that promote esports betting and skin gambling to players and their social networks of other players, friends, and family members (Greer et al., 2019; King, 2018). Some video gamers may be motivated towards skin gambling as a strategy to obtain skins for their collection or to exchange for money or something else of value. There are mixed findings as to whether playing video games is directly associated with video game-related gambling (Abarbanel et al., 2020; Macey & Hamari, 2018b; Macey et al., 2020; Wardle et al., 2020). Wardle and colleagues (2020) found 16–24-year-old British youth were more likely to bet on esports when heavily involved in playing video games compared to sports/event bettors and non-gamblers. In a sample of adult past year video gamers, esports bettors were more likely than non-esports bettors to play video games once a week and to play more game genres (Abarbanel et al., 2020). In contrast, studies by Macey and colleagues found either no relationship between video gaming consumption and esports betting (2020), or a small positive association between gaming and video game-related gambling (2018b). Such findings should be contextualised within other evidence that indicates that video gaming can positively impact on well-being (Johannes et al., 2021; Przybylski & Mishkin, 2016). This preliminary evidence suggests that playing certain video games may facilitate video game-related gambling, but the relationship between gaming involvement and gambling-like activities is likely to depend on many other variables.

*Buying skins and/or loot boxes.* Referencing Fig. 1, based on emerging evidence it is predicted in path A that *buying skins or loot boxes* will be positively associated with *esports skin betting* and *skin gambling on games of chance*. As the monetisation strategies of the gaming industry have increased over the last decade, so too has the prevalence of microtransactions, with the most common types of purchases being skins and loot boxes (Zendle et al., 2020a, 2020b). Loot boxes contain randomly rewarded digital items, including skins. While hundreds of games offer skins, it is mainly those developed by the Valve Corporation (i.e., CSGO, DOTA2, PUBG) that can be used for gambling via third party websites (Greer et al., 2019; King, 2018). We suggest that individuals purchasing skins and loot boxes are more likely to be exposed to opportunities to use skins to bet on esports or games of chance, whether it be via watching online influencers promoting these websites, digital marketing, or referrals from peers/friends (Greer et al., 2019; King, 2018; Parent Zone, 2018). Macey and Hamari (2018a) found an association between greater monetary spending on loot boxes and involvement in skin gambling, with loot box purchasers twice as likely to use skins to gamble than those opening loot boxes for free. Rockloff and colleagues (2020) asked about motivations for purchasing loot boxes, with 21.3% of adolescent (12–17) and 27.3% of young adults (18–24) motivated by obtaining skins for gambling. Research also shows a relationship between loot box purchasing and esports betting amongst Australian adolescents (Hing et al., 2020) and emerging youth in Great Britain (Wardle et al., 2020). Lastly, we acknowledge that loot box purchasing itself has been considered by some academics as a video game-related gambling activity and found to be linked to gambling problems (Brooks & Clark, 2019; Drummond & Sauer, 2018; King & Delfabbro, 2019; Li et al., 2019; Rockloff et al., 2021; Zendle & Cairns, 2018, 2019). However, the current paper excludes loot box purchasing as a gambling activity because it is not regulated as gambling in most countries, instead focusing on loot box purchasing as a mechanism for obtaining skins for gambling.

*Esports viewing.* In path A we predict that e*sports viewing* will be positively associated with *esports cash betting* and *esports skin betting*. Greater consumption of watching esports is likely to lead to greater exposure and encouragement to engage in esports betting, as influenced mainly by gambling marketing, online influencers, and social networks (Abarbanel & Johnson, 2020; Abarbanel & Phung, 2019; Ipsos MORI, 2020; Kelly & Gerrish, 2019; VicHealth, 2020). Gambling marketing is present in esports (Kelly & Gerrish, 2019; Wardle, 2021) and appears to be targeting young people (Ipsos MORI, 2020). Furthermore, Abarbanel and Phung (2019) found that adult video gamers were more likely to recall seeing and to approve of gambling advertising in esports if they had watched or bet on esports in the last 12 months. These findings suggest a relationship between esports viewing and esports betting, as influenced by gambling marketing in esports. Research has found that greater esports viewership (i.e., frequency, time spent, different types watched) was associated with betting on esports (Abarbanel et al., 2020; Macey et al., 2020) and more frequent betting (Macey & Hamari, 2018a). Macey and Hamari (2018a) also found a strong positive relationship between watching esports and video game-related gambling, which included esports betting and skin gambling.

*Playing esports.* No previous research has explored the relationship between playing competitive esports and esports betting. Younger video gamers are more likely than older players to seek out a career as a professional esports player (Bányai et al., 2020), where esports events are occurring at local, amateur, and professional levels. In addition, research with sports and fantasy sports athletes at college/university and professional levels finds high rates of gambling and problem gambling (see reviews by Derevensky et al., 2019; Winters & Derevensky, 2019). In line with other types of athletes, esports professional athletes—and even emerging amateur competitors–may likewise be attracted gambling such as esports betting. Path A of the model will test whether *esports playing* is positively associated with *esports cash betting* or *esports skin betting*.

### Bidirectional Pathway Between Video Game-Related Gambling and Traditional Gambling (B, C)

Studies have shown correlational, but not causal, relationships between video game-related gambling and traditional gambling. Esports bettors and skin gamblers appear to have high levels of involvement in traditional forms of gambling (Gainsbury et al., 2017a, 2017b; Greer et al., 2021; Lelonek-Kuleta & Bartczuk, 2021; Wardle, 2019; Wardle et al., 2020). In addition, video game-related gambling is positively associated with online gambling (Macey & Hamari, 2018a) and esports betting with greater gambling consumption (Macey et al., 2020). The second component of Fig. 1 captures our proposition that the relationship between video game-related gambling and traditional gambling is bidirectional, in that greater involvement in one increases the likelihood of involvement in the other.

Path B in Fig. 1 proposes that involvement in video game-related gambling leads to commercial or “traditional” forms of gambling, especially those that are structurally similar to the gambling activities in video games (e.g., sports betting, casino table games, EGMs). It is proposed in Path B that the consumption of e*sports cash betting*, *esports skin betting*, and s*kin gambling on games of chance* will be positively associated with *traditional gambling*. Underlining this pathway is evidence that esports betting and skin gambling are attracting young and predominantly male consumers (Browne et al., 2019; Gainsbury et al., 2017a; Gambling Commission, 2019a, 2019b; Greer et al., 2021; Wardle et al., 2020), two demographic risk factors for traditional gambling involvement and harm (see reviews by Abbott et al., 2018; Miller, 2015). Hypothetically, greater involvement in esports betting and/or skin gambling by this vulnerable group could lead to the encouragement and normalisation of gambling and in turn facilitate the transition to traditional gambling, especially as they reach legal gambling age. Given that esports betting and skin gambling only emerged in the 2010s, individuals transitioning through this pathway may not be common. However, it warrants investigation because skin gambling websites remain unregulated and easily accessible to children and adolescents in most jurisdictions. Furthermore, esports betting and skin gambling websites are increasingly offering alternative forms of currency to bet with that are not regulated. For example, cryptocurrency and Blockchain technology-based currencies such as non-fungible tokens (NFTs) are alternatives to cash for betting such as VGO skins (Abarbanel & Macey, 2018; Gainsbury & Blaszczynski, 2017).

Path C in Fig. 1 is premised on the assumption that involvement in traditional forms of gambling leads to video game-related gambling. We expect that the consumption of *traditional gambling* will be positively associated with the consumption of e*sports cash betting*, *esports skin betting*, and *skin gambling on games of chance*. As mentioned above, given esports betting and skin gambling are emerging gambling activities, it is more likely that traditional gambling involvement will precede these activities for adult respondents. By adulthood most people will have tried gambling in some form. However, we argue that highly engaged traditional gamblers will have been more likely to take up esports betting and/or skin gambling as they became available to them. Participation in particular gambling activities may differentially facilitate uptake of the three activities. For example, a sports bettor could be exposed to esports cash betting marketing via their sports betting operator. On the other hand, a gambler who usually spends money on EGMs or casino table games may decide to try betting with skins on games of chance resembling these activities.

### The Impacts: Problem Gambling and Gambling-Related Harm (D, E)

The final pathways in Fig. 1 (Paths D & E) are based on the premise that for video game-related gamblers their gambling involvement, particularly with the addition of traditional gambling and greater gambling intensity, could lead to problem gambling and/or gambling-related harm. The impacts of traditional forms of gambling are well established, but less is known about how much harm esports betting and skin gambling cause to the gambler. Evidence is emerging that shows a relationship between engagement in esports betting and skin gambling and being at risk for gambling problems (Browne et al., 2019; Hing, Russell, et al., 2021; Greer et al., 2021; Macey & Hamari, 2018a; Macey et al., 2020; Marchica et al., 2021; Wardle, 2019; Zendle, 2020). High levels of gambling problems have been found in samples of adult esports bettors (Browne et al., 2019; Gainsbury et al., 2017b; Greer et al., 2021; Lelonek-Kuleta & Bartczuk, 2021), adolescent esports bettors (Marchica et al., 2021), esports bettors aged 16–24 years (Wardle et al., 2020), adolescent past-month skin gamblers (Hing, Russell, et al., 2021), and children betting with skins (Wardle, 2019). Other research shows that gambling problems and/or harm are associated with greater consumption of video game-related gambling (Macey & Hamari, 2018a), being an esports bettor (Gainsbury et al., 2017b; Greer et al., 2021; Wardle et al., 2020), being a skin gambler (Wardle, 2019), and more frequent esports betting (Gainsbury et al., 2019; Rockloff et al., 2020; Russell et al., 2020; Zendle, 2020) and skin gambling (Greer et al., 2021).

Given esports bettors and skin gamblers typically participate in other forms of gambling, a mere association between esports and gambling problems is weak evidence for a direct causal link. The question therefore becomes for these esports bettors and skin gamblers: to what degree is video game-related gambling directly harmful when controlling for levels of traditional gambling? Few studies have examined the unique contribution of esports betting and skin gambling to gambling problems or harm (Browne et al., 2019; Gainsbury et al., 2019; Greer et al., 2021; Wardle, 2019). In a sample of regular adult esports bettors also highly involved in traditional gambling, Greer and colleagues (2021) found that only more frequent esports skin betting and skin gambling on games of chance were significantly predictive of problem gambling severity. Other research has found skin gambling (Wardle, 2019) and esports betting (Browne et al., 2019; Gainsbury et al., 2019) were no longer associated with gambling problems or harm when accounting for participation in other gambling activities. However, these studies were conducted with broader samples (i.e., online gamblers, general population) where the sample sizes of esports bettors and skin gamblers were very small. The current study will attempt to replicate the work of Greer et al. (2021) with the current sample of esports bettors and skin gamblers to determine how much video game-related gambling uniquely contributes to gambling problems and harm.

### Research Aims

The aim of this research was to test the conceptual relationships between video gaming involvement, video game-related gambling, traditional gambling, and impacts of gambling, as guided by four research questions: (1) are video game behaviours associated with greater frequency of esports betting or skin gambling? (Path A); (2) does greater frequency of esports betting or skin gambling increase the likelihood of involvement in traditional forms of gambling? (Path B); (3) does greater involvement in traditional gambling activities increase the likelihood of involvement esports betting or skin gambling? (Path C); and (4) how much gambling-related harm, if any, do esports bettors and skin gamblers experience, and how much is attributable to video game-related gambling (Path D) versus traditional gambling (Path E)?

## Methods

### Participants and Procedure

An online survey of esports bettors and skin gamblers was conducted between October 2018 and February 2019. Recruitment was via: 1) Amazon's Mechanical Turk (an online crowdsourcing panel) with participants each paid US$1.80 compensation (n = 589, 79.9%), and 2) social media posts (Facebook, Twitter, Reddit) targeted to online communities for video-gaming, esports, esports betting, skins and skin gambling, and video gaming (n = 148, 20.1%). Participants sourced via social media could enter a prize draw to win one of five US$50 Amazon Gift Cards, or 1 × Samsung Galaxy Tablet. Given the very low prevalence of participation in these emerging gambling activities in adult populations (i.e., less than 1%: Browne et al., 2019; Hing, Browne, et al., 2021), multiple recruitment sources were required to achieve a large enough sample size for robust data analyses. The survey sampled individuals who had gambled in the *last 6 months* on either: esports cash betting, esports skin betting, or skin gambling on games of chance. Countries sampled were the USA (n = 538), UK (n = 119), Canada (n = 71), and Ireland (n = 9). Respondents answered an attention check question early in the survey and were screened out if they selected an incorrect response.

The rate of completes (n = 737) for persons starting the survey (n = 2,952) was 25.0%. The remainder of the sample was excluded for the following reasons: ineligible as they did not bet on any of the three activities of interest (25.9%), incomplete survey (21.8%), failed attention check (8.4%), ineligible country (8.3%), duplicate response (7.2%), poor data quality (1.8%), under 18 years of age (1.2%), and no consent (0.5%). The final sample comprised 737 participants (80.2% male) ranging between 18 and 64 years of age (*M* = 28.97 years, *SD* = 8.07). Table 1 provides demographic and descriptive statistics for the final sample.

**Table 1:** Demographic characteristics of the final sample

Variable	n = 737 (n, %)
*Age (scale)*	Mean = 28.97 years (*SD* = 8.07)
*Gender*	
Male	591 (80.2)
Female	146 (19.8)
*Country of residence*	
USA	538 (73.0)
UK	119 (16.1)
Canada	71 (9.6)
Ireland	9 (1.2)
*Marital status*	
Single, never married	445 (60.4)
Married/domestic partnership	269 (36.5)
Divorced/separated / widowed	23 (3.1)
*Highest level of education*	
Primary school	39 (5.3)
Secondary school	115 (15.6)
Post-secondary/tertiary	148 (20.1)
Bachelor/master/doctoral	435 (59.0)
*Employment status*	
Employed	631 (85.6)
Unemployed	106 (14.4)
*Annual personal income*	
$0—$19,999 per year	183 (24.8)
$20,000—$39,999 per year	175 (23.7)
$40,000—$74,999 per year	242 (32.8)
$75,000—$149,999 per year	93 (12.6)
$150,000 or more per year	13 (1.8)
Prefer not to say	31 (4.2)

Participants reported in the last 6 months having engaged in one or more of the following: 1) esports cash betting (n = 576, 78.2%), 2) esports skin betting (n = 184, 25.0%), and/or 3) skin gambling on games of chance (n = 330, 44.8%). There was considerable overlap between these groups. The largest subset engaged in only esports cash betting (45.7%), compared to a smaller proportion of ‘skin gamblers only’ (12.2%) and ‘esports skin bettors only’ (3.4%). Some of the sample who engaged in esports cash betting also engaged in skin gambling (17.1%), both esports skin betting and skin gambling (9.2%), or esports skin betting (6.1%). Finally, 6.2% of the sample used skins to bet on both esports and games of chance. The most common games of chance bet on with skins were roulette (30.7%), coinflip (21.6%), jackpot (19.6%), and blackjack (14.1%)–other games included case openings, dice, crash game, tradeup game, cards, slots, and mines (all < 12%).

### Measures

#### Video Game Involvement

Data for video gaming and esports viewing were collected on lifetime participation (no, yes), and if ‘yes’, the frequency in the last 6 months. Frequency was coded as never (0), more than 6 months ago (1), at least 6 monthly (2), at least fortnightly (3), and at least weekly (4). Last 6-month expenditure on video gaming purchases of skins and loot boxes was collected (no, yes). Playing esports competitively was measured by asking if they had *ever* competed in an esports event (0 = no, 1 = yes) as a: professional player for a financial prize; amateur player for a financial prize; and/or a player in a friendly tournament with no financial prize (e.g., LANs). Table 2 shows descriptive statistics of video gaming and esports consumption.

**Table 2:** Video gaming and esports consumption (n = 737)

Variable	n = 737 (n, %)
*Frequency of video gaming*	
At least weekly	637 (86.4)
At least fortnightly	20 (2.7)
At least monthly	28 (3.8)
At least 6 monthly	19 (2.6)
More than 6 months ago	23 (3.1)
Never	10 (1.4)
*Video game purchases—last 6 months (yes)*	
Skins	470 (66.8)
Loot boxes	332 (47.2)
*Frequency of esports viewing*	
At least weekly	355 (48.2)
At least fortnightly	105 (14.2)
At least monthly	70 (9.5)
At least 6 monthly	16 (2.2)
More than 6 months ago	42 (5.7)
Never	
*Esports playing by type of competition—ever (yes)*	
Professional player, for a financial prize	41 (5.6)
Amateur player, for a financial prize	154 (20.9)
Player in a friendly tournament with no financial prize (e.g., LANs)	274 (37.2)

#### Video Game-Related and Traditional Gambling Activities

Gambling participation was collected for 12 activities, three ‘video game-related gambling’ activities and nine ‘traditional’ gambling activities, seven of which were selected for analysis: esports cash betting, esports skin gambling, skin gambling on games of chance, electronic gaming machines (EGMs), casino table games, sports betting, and fantasy sports (see Table 3). Participants were identified as participating in esports cash betting if they bet on esports using money (debit or credit), cryptocurrency, or purchased virtual currency (e.g., coins) with money. Betting with skins on esports (esports skin betting) or games of chance (skin gambling) included using skins deposited via Steam, depositing VGO skins, or purchasing the gambling website’s virtual currency (e.g., coins) with skin deposits. Gambling frequency for all activities was recoded as: never/not in the last 6 months (0), at least 6 monthly (1), at least monthly (2), at least fortnightly (3), and at least weekly (4).

**Table 3:** Gambling frequency by activity (n = 737)

Frequency by activity, n (%)	Weekly	Fortnightly	Monthly	Six Monthly	Less than six monthly	Never
*Video game-related gambling activities*						
Esports cash betting	138 (18.7)	76 (10.3)	164 (22.3)	198 (26.9)	11 (1.5)	150 (20.4)
Esports skin betting	42 (5.7)	26 (3.5)	44 (6.0)	72 (9.8)	39 (5.3)	514 (69.7)
Skin gambling (games of chance)	71 (9.6)	38 (5.2)	90 (12.2)	131 (17.8)	33 (4.5)	374 (50.7)
Traditional gambling activities*						
EGMs	26 (3.5)	33 (4.5)	71 (9.6)	183 (24.8)	79 (10.7)	345 (46.8)
Casino table games	41 (5.6)	40 (5.4)	78 (10.6)	198 (26.9)	72 (9.8)	308 (41.8)
Sports betting	131 (17.8)	32 (4.3)	69 (9.4)	88 (11.9)	28 (3.8)	389 (52.8)
Fantasy sports	67 (9.1)	20 (2.7)	40 (5.4)	68 (9.2)	24 (3.3)	518 (70.3)

^*^Excluded traditional gambling activities: private betting for money, horse/greyhound wagering, Keno, lotteries and scratch tickets, and bingo

#### Problem Gambling and Gambling-Related Harm

Problem gambling was measured using the Problem Gambling Severity Index (PGSI: Ferris & Wynne, 2001) for the last 6-month timeframe. The PGSI consists of 9-items rated on a 4-point rating scale: ‘never’ (0), ‘sometimes’ (1), ‘most of the time’ (2), and ‘almost always’ (3). Total scores range from 0 to 27 categorising gamblers by score into: non-problem (0), low-risk (1–2), moderate-risk (3–7), and problem gamblers (8–27). The 10-item Short Gambling Harms Screen (SGHS, Browne et al., 2017) was used to measure gambling-related harm experienced from all gambling, over the last 6 months. Participants answered to experiencing each gambling-related harm (0 = no, 1 = yes), with total scores ranging from 0 to 10 and categorised into groups: 0 harms, 1–2 harms, 3–4 harms, and 5–10 harms. Table 4 shows descriptive statistics for PGSI and SGHS in the sample.

**Table 4:** PGSI and SGHS categorisation and mean scores (n = 737)

Variable	n = 737 (n, %)
*Problem gambling severity index (PGSI)*	
Non-problem (0)	129 (17.5)
Low risk (1–2)	165 (22.4)
Moderate risk (3–7)	219 (29.7)
Problem (8 +)	224 (30.4)
*Mean (SD)*	*5.90 (6.15)*
*Short gambling harms screen (SGHS)*	
0 harms	240 (32.6)
1–2 harms	175 (23.7)
3–4 harms	97 (13.2)
5–10 harms	225 (30.5)
*Mean (SD)*	*2.98 (3.09)*

### Statistical Analysis

The theoretical model was tested using a series of ordinal regressions given the dependent variables in each path were measures using ordinal scales. Given the moderate sample size, we opted not to undertake a formal path analytic or structural evaluation of the model structure. Thus, the statistical analysis should be understood in terms of testing a set of relationships linked by a common theoretical model. The following variables were transformed for analyses: (a) video gaming frequency into a dichotomous variable (0 = at least weekly, 1 = less than weekly) given that 86.4% of participants played video games at least weekly; (b) esports viewing, all video game-related gambling variables, and all traditional gambling variables: six to five point scale from 0 to 4, combining ‘less than monthly’ and ‘never’; (c) professional and amateur esports playing variables combined: 0 = no, 1 = yes; and (d) PGSI and SGHS scores into categories as per Table 4. In addition, an ‘Any traditional gambling at least monthly’ (0 = no, 1 = yes) was computed from frequency of the four traditional gambling activities: EGMs, casino table games, sports betting, and fantasy sports. Analyses were conducted with variables as continuous variables showing the same results as using categorical variables, therefore categorical variables were reported for easier interpretation by the reader.

Thirteen ordinal regressions were conducted to test each path of the conceptual model: (a) Path A: three regressions with esports cash betting, esports skin betting, and skin gambling as dependent variables (DVs) and video gaming frequency, esports viewing frequency, purchasing skins, purchasing loot boxes, playing esports (no financial prize), and playing esports (financial prize) are independent predictor variables (IVs); (b) Path B: five regressions with the EGM frequency, casino table game frequency, sports betting frequency, fantasy sports frequency, and any traditional gambling at least monthly entered as DVs, and frequency of the three video-game related gambling activities as IVs; (c) Path C: three regressions with esports cash betting, esports skin betting, and skin gambling as DVs and EGM frequency, casino table game frequency, sports betting frequency, fantasy sports frequency as IVs; (d) Path D and E: two ordinal regressions with PGSI categories and SGHS categories as DVs, and the three video game-related gambling frequency and four traditional gambling frequency variables entered as IVs. Non-parametric Spearman’s rho correlations were conducted to test the relationships between variables in the model (see Supplementary Table 1).

Prior to the main statistical analyses being conducted, preliminary analyses were conducted to identify any differences in the sample by the two recruitment sources, Mechanical Turk and social media. While differences were found in the demographics and levels of engagement in video gaming and video game-related gambling activities (i.e., sample recruited via social media were younger, more engaged), the samples had similar levels of involvement in traditional gambling and levels of gambling problems/harm. Taking this into consideration, the authors concluded that sampling from these multiple sources provided a better coverage of esports bettors/skin gamblers and decided to run the statistical analyses on the combined sample rather than separately.

## Results

### Path A: Video Game Involvement Associations with Video Game-Related Gambling

Of the six video gaming activities, purchasing skins was associated with greater frequency of esports skin betting and skin gambling on games of chance, but lower frequency of esports cash betting. More frequent esports viewing predicted more frequent esports cash betting, but not esports skin betting (Table 5).

**Table 5:** Path A: Ordinal regressions of video game activities predicting video game-related gambling (N = 737)

Video game-related gambling (DVs)												
	Esports cash betting frequency				Esports skin betting frequency				Skin gambling frequency			
	Odds Ratio	Confidence Intervals		Cor.	Odds Ratio	Confidence Intervals		Cor.	Odds Ratio	Confidence Intervals		Cor.
Video game activity variables (IVs)		Lower	Upper			Lower	Upper			Lower	Upper	
Video gaming frequency	0.694	0.472	1.021	− .059	1.780	0.929	3.413	.114**	1.300	0.807	2.094	.114**
Esports viewing frequency	1.433***	1.283	1.601	.220***	1.043	0.900	1.209	.094**	0.991	0.880	1.117	.076*
Purchased skin last 6 months	0.658*	0.480	0.903	− .096**	2.111**	1.353	3.293	.190***	3.588**	2.479	5.194	.311***
Purchased loot boxes last 6 months	0.820	0.602	1.117	− .058	1.226	0.840	1.789	.139***	1.213	0.882	1.669	.186***
Esports played ever, no financial prize	0.824	0.623	1.089	− .019	1.369	0.966	1.941	.112**	0.848	0.626	1.147	.020
Esports played ever, financial prize	1.313	0.947	1.820	.091*	1.031	0.692	1.535	.042	1.025	0.726	1.448	.040
Statistics	LR χ^2^ = 58.06, df = 6, *p* < 0.001, Pseudo R^2^ = 0.079				LR χ^2^ = 38.91, df = 6, *p* < 0.001, Pseudo R^2^ = 0.062				LR χ^2^ = 80.43, df = 6, *p* < 0.001, Pseudo R^2^ = 0.112			

*p < 0.05; **p < 0.01; ***p < 0.001; Cor = Spearman’s rho correlation. Video gaming frequency (0 = less than weekly; 1 = at least weekly); Esports viewing, esports cash betting, esports skin betting, and skin gambling frequency ordinal scales (0 = never/more than 6 months ago to 4 = at least weekly); purchasing skins, loot boxes, esports playing with and without a financial prize all (0 = no; 1 = yes); Pseudo R^2^ = Nagelkerke

### Path B: Video Game-Related Gambling Influencing Traditional Gambling

As per Table 6, only esports cash betting frequency significantly predicted greater frequency of individual traditional gambling activities (EGMs, casino table games, sports betting, and fantasy sports betting) as well as regular gambling on at least one of four gambling activities. Conversely, higher levels of skin gambling on games of chance were associated with lower frequency of sports betting. Esports skin betting frequency was not associated with any of the measured traditional gambling activities but was correlated with skin gambling frequency (r_s_ = 0.229, *p* < 0.001).

**Table 6:** Path B: Ordinal regressions of video game-related gambling predicting traditional gambling (N = 737)

Traditional gambling (DVs)															
Video game-related gambling variables (IVs)	EGM freq			Casino table game freq			Sports betting freq			Fantasy sports betting freq			Any traditional gambling at least monthly (no, yes)		
	Odds Ratio	Confidence Intervals		Odds Ratio	Confidence Intervals		Odds Ratio	Confidence Intervals		Odds Ratio	Confidence Intervals		Odds Ratio	Confidence Intervals	
		Lower	Upper		Lower	Upper		Lower	Upper		Lower	Upper		Lower	Upper
Esports cash betting freq	1.154**	1.043	1.278	1.261***	1.141	1.393	1.767***	1.579	1.978	1.209**	1.076	1.358	1.589***	1.418	1.780
Esports skin betting freq	1.025	0.896	1.174	1.075	0.945	1.223	1.060	0.922	1.218	0.960	0.825	1.117	1.042	0.902	1.203
Skin gambling freq	0.994	0.887	1.114	0.961	0.860	1.074	0.805**	0.712	0.910	1.059	0.935	1.201	0.984	0.871	1.112
Statistics	LR χ^2^ = 7.84, df = 3, p = 0.049, Pseudo R^2^ = 0.012			LR χ^2^ = 22.49, df = 3, p < 0.001, Pseudo R^2^ = 0.033			LR χ^2^ = 116.23, df = 3, p < 0.001, Pseudo R^2^ = 0.159			LR χ^2^ = 11.03, df = 3, p = 0.012, Pseudo R^2^ = 0.018			LR χ^2^ = 70.39, df = 3, p < 0.001, Pseudo R^2^ = 0.121		

*p < 0.05; **p < 0.01; ***p < 0.001; Freq = frequency, all ordinal scales (0 = never/more than 6 months ago to 4 = at least weekly); Pseudo R^2^ = Nagelkerke. Spearman’s rho correlation coefficients on significant predictors: esports cash betting frequency to EGM frequency = .106**; esports cash betting frequency to casino table game frequency = .169***; esports cash betting frequency to sports betting frequency = .344***; esports cash betting frequency to fantasy sports betting frequency = .121**; esports cash betting frequency to any traditional gambling at least monthly = .306***; Skin gambling frequency to sports betting frequency =  − .151***

### Path C: Traditional Gambling Influencing Video Game-Related Gambling

Non-parametric correlations (Table 7) showed significant positive relationships between esports cash betting frequency and all four traditional gambling activities. However, when factoring in all traditional gambling activities only greater frequency of sports betting predicted greater frequency of esports cash betting. Inversely, greater sports betting frequency predicted lower skin gambling on games of chance. None of the entered traditional gambling activities were predictive of esports skin betting frequency.

**Table 7:** Path C: Ordinal regressions of traditional gambling predicting video game-related gambling (N = 737)

Video game-related gambling (DVs)												
Traditional gambling variables (IVs)	Esports cash betting frequency				Esports skin betting frequency				Skin gambling frequency			
	Odds Ratio	Confidence Intervals		Cor	Odds Ratio	Confidence Intervals		Cor	Odds Ratio	Confidence Intervals		Cor
		Lower	Upper			Lower	Upper			Lower	Upper	
EGM freq	0.986	0.848	1.146	.106**	1.061	0.881	1.277	− .012	1.109	0.946	1.301	− .007
Casino table games freq	1.098	0.957	1.260	.169***	1.010	0.851	1.199	− .001	1.032	0.893	1.193	− .016
Sports betting freq	1.627***	1.474	1.796	.344***	0.938	0.830	1.059	− .045	0.789***	0.709	0.878	− .151***
Fantasy sports betting freq	1.008	0.901	1.129	.121**	0.921	0.790	1.073	− .040	1.085	0.959	1.227	.015
Statistics	LR χ^2^ = 124.44, df = 4, *p* = < 0.001, Pseudo R^2^ = 0.162				LR χ^2^ = 3.25, df = 4, *p* = 0.517, Pseudo R^2^ = 0.005				LR χ^2^ = 20.48, df = 4, *p* = < 0.001, Pseudo R^2^ = 0.030			

*p < 0.05; **p < 0.01; ***p < 0.001; Cor = Spearman’s rho correlation; Freq = frequency, all ordinal scales (0 = never/more than 6 months ago to 4 = at least weekly); Pseudo R^2^ = Nagelkerke

### The Relative Impacts of Video Game-Related Gambling (Path D) and Traditional Gambling (Path E)

Overall, the sample of esports bettors and skin gamblers experienced high levels of problem gambling severity (PGSI: mean 5.90, 82.5% at-risk) and gambling-related harm (SGHS: mean 2.98, 67.4% at least one harm). PGSI and SGHS were highly correlated (r_s_ = 0.670, *p* < 0.001). As shown in Table 8, when factoring in involvement in all gambling activities only greater gambling frequency in three activities significantly contributed to both being a higher at-risk gambler (PGSI) and experiencing greater gambling-related harm (SGHS): skin gambling on games of chance (path D), electronic gaming machines (path E), and sports betting (path E).

**Table 8:** Path D and E: Ordinal regressions video game-related gambling and traditional gambling activities predicting PGSI and SGHS (N = 737)

Gambling impact (DVs)								
Gambling activities (IVs)	Problem Gambling Severity (PGSI 4 category)				Number of Gambling Harms (SGHS 4 category)			
	Odds Ratio	Confidence Intervals		Cor	Odds Ratio	Confidence Intervals		Cor
		Lower	Upper			Lower	Upper	
*Video game-related gambling variables (Path D)*								
Esports cash betting freq	1.033	0.933	1.143	.061	0.999	0.903	1.105	.036
Esports skin betting freq	0.952	0.840	1.080	.007	1.012	0.893	1.148	.024
Skin gambling freq	1.322***	1.185	1.476	.138***	1.170**	1.052	1.301	.076*
*Traditional gambling variables (Path E)*								
EGMs freq	1.234**	1.062	1.435	.093*	1.178*	1.011	1.372	.080*
Casino table games freq	1.097	0.959	1.255	.094*	1.114	0.973	1.276	.105**
Sports betting freq	1.135*	1.027	1.254	.105**	1.122*	1.014	1.241	.087*
Fantasy sports betting freq	0.931	0.831	1.042	− .001	0.920	0.820	1.032	.004
*Statistics*	LR χ^2^ = 54.45 df = 7, p < 0.001, Pseudo R^2^ = 0.076				LR χ^2^ = 30.77, df = 7, p < 0.001, Pseudo R^2^ = 0.044			

*p < 0.05; **p < 0.01; ***p < 0.001; Cor = Spearman’s rho correlation; PGSI = Problem Gambling Severity (0 = non-problem, 1 = low-risk, 2 = moderate-risk, 3 = problem); SGHS = Short Gambling Harm Screen (0 = 0 harms, 1 = 1–2 harms, 2 = 3–4 harms, 3 = 5–10 harms); Freq = Frequency = all ordinal scales (0 = never/more than 6 months ago to 4 = at least weekly); Pseudo R^2^ = Nagelkerke

## Discussion

**Fig. 2:**
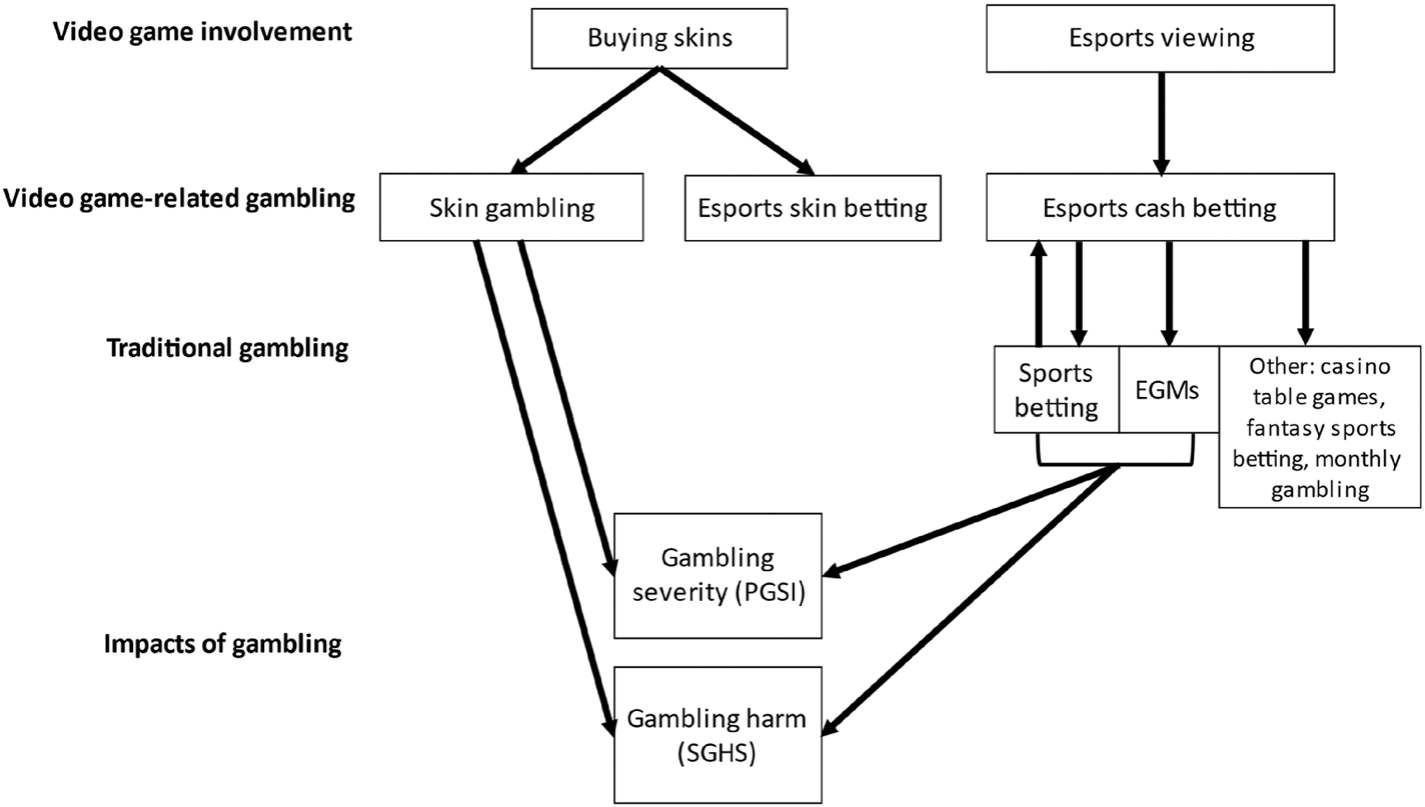
Revised conceptual model of the relationships between video game-related gambling and video game involvement, traditional gambling, and the impacts of gambling

The aim of this research was to examine relationships between video gaming involvement, video game-related gambling, traditional gambling, and impacts to the gambler, as guided by a conceptual framework. Explored in a sample of recent esports bettors and skin gamblers, the conceptual model was not supported in its entirety. Rather the results demonstrated what may be two distinctly different pathways surrounding esports cash betting and skin gambling, which are depicted in a revised conceptual model in Fig. 2 (i.e., where participation and/or frequency in one activity is positively associated with participation and/or frequency in another activity).

### Skin Gambling is Uniquely Associated with At-Risk Gambling and Harm

The finding that *purchasing skins* predicted greater gambling with skins on games of chance (*skin gambling*) and *esports skin betting* was not surprising, considering skins are necessary for gambling on these activities. Neither skin gambling nor esports skin betting were shown to be associated with greater frequency of traditional gambling, even for activities which are arguably structurally similar (e.g., casino table games, sports betting). The strongest finding was that *skin gambling on games of chance*, but not esports skin betting, uniquely contributed to being at *greater risk of problem gambling* (PGSI) *and experiencing gambling-related harm* (SGHS) when factoring in traditional gambling. That is, using skins as currency for more traditional forms of gambling was related to gambling problems and harm rather than betting specifically on esports. Thus, skins used for games of chance may simply be an accessible form of currency for these gamblers and is the source of their gambling-harm. An alternative explanation is that the games of chance used for skin gambling (e.g., roulette, coinflip, slots) are more likely to lead to gambling problems and harm due to their structural characteristics similar to casino table games and EGMs which are known to facilitate persistence, loss chasing and impaired control (Currie et al., 2021; Dowling et al., 2005; Schull, 2012; Mazar et al., 2020; Williams et al., 2021). In contrast, esports betting, whether using cash or skins, is based on discrete events that do not provide the opportunity for continuous gambling. This finding would be consistent with Greer et al. (2021) who found in an Australian sample of esports cash or skin bettors, that both esports skin betting and skin gambling predicted greater PGSI. In contrast, a secondary analysis of British adolescents (11–16 years) found that skin gamblers were at greater risk for gambling problems than non-skin gamblers, but skin gambling was not significantly associated with at-risk gambling when controlling for traditional gambling (Wardle, 2019). The difference in findings could be due to how “skin gambling” was operationalised. The current research and that by Greer and colleagues (2021) defined skin gambling as within the last 6 months using skins or skin deposits, including those from Steam and VGO items, for gambling on games of chance (e.g., coinflip, roulette, jackpot). In the current study skins were defined as “virtual in-game items, such as weapons (guns, knives, and daggers), cases, case keys, stickers, and graffiti, which offer purely cosmetic alterations to base models of these items,” and the respondents were instructed to exclude loot boxes. In Wardle’s analysis of the British Youth Gambling Survey, data on past month skin gambling was used, being defined as a “bet with in-game items for the chance to win more of them” (Wardle, 2019). Therefore, skin gambling in this British study could have included other types of in-game items than skins (e.g., virtual currency, loot boxes) and no distinction was made between the type of product bet on (i.e., esports, games of chance, other types of activities). Thus, the present study was more specific in making a distinction between betting with skins on esports versus other types of games, such as roulette. The present results suggest this distinction is important. Using skins to bet on esports may be indicative of an interest in esports, rather than more purely an interest in betting. In contrast, using skins to gamble on games of chance, can be indicative of a person who is interested in skins as a surrogate currency or obtaining skins for their own benefit (i.e., their collection, for use in a video game). Moreover, many traditional online games allow for faster-paced gambling, which in isolation facilitates larger potential losses (Abbott et al., 2018).

### Esports Cash Betting is Associated with Esports Viewing and Traditional Gambling, But Not Directly with Harm

Video gaming, playing esports competitively, or buying loot boxes do not necessarily lead to video game-related gambling in the form of esports betting (cash or skins) or skin gambling on games of chance. Perhaps unsurprisingly, respondents who reported frequently viewing esports were also more likely to bet on esports with cash more frequently. Esports viewers are exposed and encouraged towards esports betting via gambling marketing, online influencers, and social networks in these environments (Abarbanel & Johnson, 2020; Abarbanel & Phung, 2019; Ipsos MORI, 2020; Kelly & Gerrish, 2019; VicHealth, 2020; Wardle, 2021). The current findings also support recent research that greater esports viewership is associated with esports betting (Abarbanel et al., 2020; Macey et al., 2020; Macey & Hamari, 2018a). However, neither these studies nor the current study asked directly about the influence of factors within the esports viewership environment, such as knowledge of and exposure to esports betting advertisements. It is also possible that betting on esports encourages watching esports in order to track the outcomes of bets placed and to learn more about the games and competitors to inform future bets.

Additionally, the current study’s findings are consistent with a bidirectional relationship between traditional forms of cash betting and cash betting on esports (though not using skins). That is, individuals who bet with cash (and not skins) also tend to bet on a variety of traditional gambling activities. In fact, the finding that more frequent sports betting predicted higher frequency esports cash betting, but *lower* frequency skin gambling indicates that in this sample esports bettors are a distinctly different group to skin gamblers. Gambling problems and gambling harm resulting from specific gambling activities could not be marginally attributed to esports cash betting, but rather frequent betting on more traditional forms of gambling (EGMs, sports betting). This was not evidence, however, that betting on esports caused no problems or harm. Instead, problems and harm could not be distinguished from the general tendency of esports bettors to bet on many forms of gambling, including traditional games (EGMs) and sports. These findings are in line with research with online gamblers finding that when controlling for gambling frequency on a range of activities, that problem gambling was only positively associated with gambling on EGMs and sports betting (Gainsbury et al., 2019). To our knowledge there is no evidence to date that esports cash betting exclusively causes gambling problems or harm, since people who bet with cash on esports tend to also bet on other traditional sports and games that could instead be the cause of their difficulties (Browne et al., 2019; Gainsbury et al., 2017b; Greer et al., 2021; Lelonek-Kuleta & Bartczuk, 2021; Wardle et., 2020). In fact, online wagering operators offer traditional esports betting alongside traditional sports betting, thereby potentially attracting highly engaged gamblers on other forms of betting.

### Limitations

The present study has limitations that warrant consideration. First, only three video game-related gambling activities were explored in the conceptual model. The authors acknowledge other converging gaming-gambling products associated with traditional gambling involvement and gambling problems/harm that could be explored in future research (i.e., purchasing loot boxes, social casino games). Due to the cross-sectional nature of the study, any associations found between variables do not necessarily infer causality, and there may be other factors not measured which influence pathways into and from video game-related gambling (i.e., motivations, individual differences). The explored relationships were restricted to the implied paths of the conceptual model, and a different model would have tested for different relationships (i.e., a path from video game-related gambling to video game involvement). In addition, the limited sample size did not allow for a path analysis and instead a large number of regressions were conducted. Lastly, the sample were specifically recruited to be adult esports bettors and/or skin gamblers and therefore the findings cannot be generalised to wider populations which may be exposed to these gambling activities—such as children and adolescents. In other words, the relationships tested in the proposed model and the research findings are only generalisable to adult esports bettors and skin gamblers.

## Conclusions

Research continues to explore whether and how specific types of video gaming involvement may lead to video game-related gambling, traditional gambling, and subsequent gambling problems and harm. The current study provides evidence that skin gambling and esports cash betting activities have distinctly different relationships to traditional gambling involvement, problems, and harm. Skin gambling stands alone as an emerging and mostly unregulated activity which does not necessarily lead to involvement in traditional gambling but contributes directly to gambling problems and harms. Esports cash betting is attracting both esports viewers and sports bettors and is associated with heavier involvement in traditional gambling. However, esports cash betting does not uniquely contribute to gambling problems and harm when controlling for traditional gambling activities. The landscapes of esports betting and skin gambling are rapidly evolving, therefore the potential risks associated with these newer forms of gambling will need continual careful attention.

## Supplementary Information

Below is the link to the electronic supplementary material.

Supplementary file1 (DOCX 18 KB)

## Data Availability

Data and material not currently available.
