# Supplementary material for: Skin Gambling Contributes to Gambling Problems and Harm After Controlling for Other Forms of Traditional Gambling
Source: J Gambl Stud. 2022 Feb 25;39(1):225–47. doi: 10.1007/s10899-022-10111-z (PMC9981708; doi:10.1007/s10899-022-10111-z)
Supplement: Supplementary file 1 — Supplementary file1 (DOCX 18 KB) [file 10899_2022_10111_MOESM1_ESM.docx]

| **Supplementary Table 1.** Spearman’s rho correlation coefficient between at-risk gambling severity (PSGI), gambling harm (SGHS), video game involvement, frequency of video game-related gambling, and frequency of traditional gambling (N = 737) | | | | | | | | | | | | | | | |
| --- | --- | --- | --- | --- | --- | --- | --- | --- | --- | --- | --- | --- | --- | --- | --- |
| Variables | PGSI | SGHS | VG | EV | Skins | LBs | ENP | EP | ECB | ESB | SG | EGM | CS | SB | FS |
| PGSI | 1 |  |  |  |  |  |  |  |  |  |  |  |  |  |  |
| SGHS | **.670***** | 1 |  |  |  |  |  |  |  |  |  |  |  |  |  |
| VG | -.001 | .057 | 1 |  |  |  |  |  |  |  |  |  |  |  |  |
| EV | **.089*** | **.080*** | **.185***** | 1 |  |  |  |  |  |  |  |  |  |  |  |
| kins | **.140***** | **.122**** | **.254***** | **.237***** | 1 |  |  |  |  |  |  |  |  |  |  |
| LBs | **.174****** | **.151***** | **.192***** | **.229***** | **.490***** | 1 |  |  |  |  |  |  |  |  |  |
| ENP | .022 | .049 | **.149***** | **.226***** | **.159***** | **.144***** | 1 |  |  |  |  |  |  |  |  |
| EP | **.119**** | **.096**** | .050 | **.235***** | **.129***** | **.158***** | **.080*** | 1 |  |  |  |  |  |  |  |
| ECB | .061 | .036 | -.059 | **.220***** | **-.096**** | -.058 | -.019 | **.091*** | 1 |  |  |  |  |  |  |
| ESB | .007 | .024 | **.114**** | **.094**** | **.190***** | **.139***** | **.112**** | .042 | **-.114**** | 1 |  |  |  |  |  |
| SG | **.138***** | **.076*** | **.114**** | **.076*** | **.311***** | **.186***** | .020 | .040 | **-.163***** | .**229***** | 1 |  |  |  |  |
| EGM | **.093*** | **.080*** | -.034 | -.039 | .042 | -.006 | .029 | -.042 | **.106**** | -.012 | -.007 | 1 |  |  |  |
| CS | **.094*** | **.105**** | -.021 | **.085*** | .018 | .027 | **.089*** | .049 | **.169***** | -.001 | -.016 | **.475***** | 1 |  |  |
| SB | **.105**** | **.087*** | -.013 | **.083*** | **-.121**** | **-.089*** | .015 | -.001 | **.344***** | -.045 | **-.151***** | **.215***** | **.286***** | 1 |  |
| FS | -.001 | .004 | -.022 | **.102**** | .024 | .024 | -.004 | .058 | **.121**** | -.040 | .015 | **.165***** | **.231***** | **.278***** | 1 |
| Relationships are significant at the *p* < 0.05 level. ** *p* < 0.01 level, *** *p* < 0.001 level. PGSI = Problem Gambling Severity (0 = non-problem, 1 = low-risk, 2 = moderate-risk, 3 = problem); SGHS = Short Gambling Harm Screen (0 = 0 harms, 1 = 1-2 harms, 2 = 3-4 harms, 3 = 5-10 harms); VG = Video gaming frequency (0 = less than weekly; 1 = at least weekly); EV = Esports viewing frequency (0 = never/more than 6 months ago to 4 = at least weekly); Skins = purchase of skins last 6 months (0 = no; 1 = yes); LBs = purchase of loot boxes last 6 months (0 = no; 1 = yes); ENP = esports player ever, no financial prize (0 = no; 1 = yes); EP = esports player ever, financial prize months (0 = no; 1 = yes); ECB= Esports cash betting frequency; ESB = Esports skin betting frequency; SG = Skin gambling on games of chance frequency; SB = Sports betting frequency; EGM = Electronic gaming machine frequency; CG = Casino table games frequency; FS = Fantasy sports betting frequency; EGM = Electronic gaming machine frequency; CG = Casino table games frequency; SB = Sports betting frequency; FS = Fantasy sports betting frequency. All frequencies 5 levels: 0 = never/more than 6 months ago to 4 = at least weekly. | | | | | | | | | | | | | | | |
